# Unlocking the potential of smart grid technologies with behavioral science

**DOI:** 10.3389/fpsyg.2015.00410

**Published:** 2015-04-09

**Authors:** Nicole D. Sintov, P. Wesley Schultz

**Affiliations:** ^1^Department of Psychology and Environmental Studies Program, Viterbi School of Engineering, Information Sciences Institute, University of Southern California, Los Angeles, CA, USA; ^2^Department of Psychology, California State University San Marcos, San Marcos, CA, USA

**Keywords:** smart grid, energy conservation, energy efficiency, behavioral science, human factors, technology adoption

## Abstract

Smart grid systems aim to provide a more stable and adaptable electricity infrastructure, and to maximize energy efficiency. Grid-linked technologies vary widely in form and function, but generally share common potentials: to reduce energy consumption via efficiency and/or curtailment, to shift use to off-peak times of day, and to enable distributed storage and generation options. Although end users are central players in these systems, they are sometimes not central considerations in technology or program design, and in some cases, their motivations for participating in such systems are not fully appreciated. Behavioral science can be instrumental in engaging end-users and maximizing the impact of smart grid technologies. In this paper, we present emerging technologies made possible by a smart grid infrastructure, and for each we highlight ways in which behavioral science can be applied to enhance their impact on energy savings.

## Background and Significance

Smart grid systems are rapidly being deployed across the world. Although smart grid technologies vary considerably, they generally share common potentials, all of which contribute to a more reliable grid: to reduce energy consumption via efficiency and/or curtailment, to shift use to off-peak times of day, and to expand distributed storage and generation options. In each of these areas, human behavior is integral to unlocking the full potentials of these smart grid technologies.

At its core, a smart grid system involves high-resolution meters for quantifying electricity consumption. However, metering infrastructure alone will not result in improved efficiency. In the 1980s, automatic meter reading (AMR) technology advanced power systems by enabling remote collection of electricity use data at higher resolution than manual readings. Building on AMR, advanced metering infrastructure (AMI) technology involves meters that collect near real-time consumption data (“smart meters”). Importantly, AMI networks also enable two-way data communication between utilities and consumers. The ability to interact with consumers in real-time is one key route for engaging consumers with techniques from behavioral science. This connectivity has spawned a variety of new programs and technologies that require consumer adoption and proper use to function optimally. Traditionally, although utilities have involved end-users to some extent in power systems, consumers have often not been central considerations in technology or program design, and in some cases, their motivations for participating in such systems have not been fully appreciated. Consequently, there is a glaring need to understand the ways in which individuals interact with smart grid systems. Leveraging behavioral science can advance our knowledge of how to partner with customers in the smart grid and ultimately lead to more efficient uses of energy.

## Behavioral Science in the Smart Grid

Historically viewed as engineering challenges, power systems have benefited from integrating behavioral science perspectives. For instance, a number of recent reviews have applied behavioral science to better understand the theoretical underpinnings of energy use behavior (Steg and Vlek, 2009), explore the effectiveness of interventions aimed at reducing energy and other resource use (Abrahamse et al., 2005; Abrahamse and Steg, 2013), identify predictors of alternative energy resource acceptance (Perlaviciute and Steg, 2014), and propose models of sustainable energy technology acceptance (Huijts et al., 2012). Building on the existing literature, this paper focuses on consumer adoption and optimal use (i.e., using the technologies in a manner that maximizes energy savings and/or peak load reductions) of emerging technologies in smart grid systems.

In the sections below, we briefly review selected models from behavioral science that aid in understanding the adoption and use of smart grid technologies. The models selected are not intended to provide an exhaustive list, but rather to illustrate some of the major conceptual and theoretical approaches that can help to inform smart grid programs. We then provide an overview of several strategies that utilize smart grid infrastructure to encourage electricity savings among residential users. We summarize specific examples, discuss the underlying behavior change tools at work, and suggest ways in which these strategies can be improved by leveraging behavioral science, offering practical advice for researchers and practitioners alike. See Table 1 for an overview.

**TABLE 1:** **Behavioral science tools for unlocking potentials of smart grid technologies**.

**Smart grid technology**	**Potential**	**Target behavior**	**Behavioral science tools**
Demand response	Reduce peak demand	Increase program enrollment	Incorporate motivators/barriers into messaging; use flexible defaults
Time-of-use pricing plans	Reduce peak demand	Increase program enrollment	Incorporate motivators/barriers into messaging
Energy feedback	Increase energy efficiency	Reduce energy consumption	Leverage social influence; tailor feedback to address barriers/motivators
Disaggregation technologies	Increase energy efficiency	Reduce energy consumption	Provide high-resolution feedback and specific recommendations
Smart automation	Reduce peak demand	Maximize participation in demand response events	Use flexible defaults
Electric vehicles	Distributed storage	Increase adoption and program enrollment	Leverage social influence and symbolic attributes; reduce barriers, including providing financial incentives; use flexible defaults
Solar panels	Distributed generation	Increase adoption	Leverage social influence and symbolic attributes; reduce barriers, including providing financial incentives

### Behavioral Foundations

There is a large and growing body of research on theoretical models that have been proposed for understanding energy use behaviors. Although a detailed theoretical synthesis is outside the scope of this paper, we selected several models that have received considerable empirical support in explaining various pro-environmental behaviors, and can be extended to better understand smart grid technology adoption and use. We link each of the models described below to one or more of the smart grid technologies discussed later in this paper:

• The Theory of Planned Behavior (TPB) postulates that behavior is proximally determined by intention to perform the behavior, which is more distally predicted by attitudes, normative beliefs, and perceived control (for details see Ajzen, 1991).
• The Norm Activation Model (NAM) posits that altruistic behavior begins with learned social norms regarding proper behavior, which give rise to personal norms tied to self-concept (Schwartz, 1994). When a person is aware of the consequences of her/his behavior, and ascribes responsibility for these consequences to the self, personal norms become “activated,” and the person will behave in accordance with them.
• The Value-Belief-Norm (VBN) theory builds on the NAM, suggesting that value orientation predicts environmental worldview, awareness of consequences, and ascription of responsibility, which in turn gives rise to norms, which more proximally predict behavior (Stern, 2000).
• Focusing more closely on social norms, The Focus Theory of Normative Conduct (Cialdini et al., 1990) differentiates between two primary types of social norms: (1) descriptive norms, which convey what others typically do in a particular situation; and (2) injunctive norms, which convey social approval or disapproval for a given behavior. The model proposes that the impact of norms on behavior depends on which norms are most salient to an individual in a given situation (e.g., Schultz et al., 2007).
• The field of Behavioral Economics also offers insights to help explain why people make decisions that do not always maximize their expected utility or economic benefit (Kahneman, 2003). This approach takes into account the influence of information processing biases on decision-making, such as choice framing effects (i.e., framing a choice as either a gain or a loss) and default policies (i.e., opt-in vs. opt-out).
• Under the framework of Self-Determination Theory, supporting an individual’s autonomy, competence, and relatedness (connection with others) fosters motivation that can increase the likelihood of engaging in a variety of behaviors (Deci and Ryan, 1985).
• The Theory of Operant Conditioning states that behavior that is reinforced or rewarded tends to be repeated, behavior that is not reinforced, and moreover, behavior that is punished, tends to become extinguished (Skinner, 1953).
• Diverging a bit from the above models, Community-Based Social Marketing (CBSM; McKenzie-Mohr, 2000) is a framework for behavior change that involves identifying motivators and barriers to the acceptance and adoption of a particular behavior among a given population, and devising tailored strategies to enhance motivators and overcome barriers. This approach has been used successfully to promote a range of pro-environmental behaviors, ranging from recycling to water efficiency to energy conservation (for detailed review of CBSM programs, see McKenzie-Mohr and Schultz, 2014). We view CBSM as one promising approach to promoting the adoption and utilization of smart grid technologies due to its flexibility and potential to address aspects of all aforementioned models.

In the remainder of this paper, we discuss how these models can be used to understand and expand the adoption and use of several smart grid technologies. Previous work has classified energy conservation behaviors into efficiency and curtailment categories (Gardner and Stern, 2008). Curtailment involves using existing equipment less frequently or intensively but requires repetition of curtailment behaviors to achieve savings. On the other hand, efficiency behaviors typically involve infrequent capital improvements and do not require the same level of repetition or behavioral maintenance. We view the smart grid technologies described below as falling into the efficiency category if they require infrequent actions on the part of the consumer and/or primarily involve utility direct control of equipment (i.e., direct control demand response, smart automation, electric vehicle adoption, and solar panel installation), whereas technologies in the curtailment category require ongoing participation by consumers to achieve energy reductions (i.e., voluntary curtailment demand response, time-of-use pricing programs, energy feedback, disaggregated feedback).

### Demand Response Programs

Electric power interruptions often result from demand exceeding available supply. Even relatively brief lapses in power reliability have significant consequences. Estimates for annual economic losses from power interruptions include €150 billion among European Union businesses and $80 billion in the United States (LaCommare and Eto, 2004). Because demand varies by time of day, growing efforts are being made to manage demand by reducing peak loads as an alternative to the traditional strategy of bringing on additional generation, usually from higher-polluting energy sources (California Independent Systems Operator, 2013). Accordingly, U.S. utilities are investing $700 million annually in demand response (DR) strategies to curtail peak loads and thereby make more efficient use of the existing generation and transmission infrastructure (United States Energy Information Administration, 2015a). Although DR forecasting models predict when, where, and how much energy will be used, solving the key problem of reducing peak demand requires programs that encourage electricity consumers to make behavioral changes.

In alignment with the curtailment vs. efficiency framework, utility DR programs generally fall into one of two categories: (1) voluntary curtailment, which involves appealing to consumers to temporarily curtail consumption by changing behavior in real-time in response to alerts (e.g., California Independent Systems Operator Flex Alerts); or (2) direct control, in which consumers permit utilities to remotely control home equipment (e.g., Southern California Edison’s air conditioning cycling program). Voluntary curtailment programs generally rely on behavioral prompts and appeals in their attempt to persuade consumers to curtail usage. However, generic informational appeals to save energy have not been particularly effective for reducing overall energy use (e.g., Schultz et al., 2007; Nolan et al., 2008; Schultz, 2010). Findings from studies of persuasion suggest alternatives for enhancing participation rates and reducing demand, for instance by tapping into social norms (Schultz et al., 2007; Nolan et al., 2008) or obtaining commitments.

Even when applying effective tools of persuasion, voluntary curtailment still relies on consumers to undertake a series of decisions and actions, including: (1) attending to the alert, (2) mentally cataloging energy use in home, (3) deciding what action(s) to take to reduce energy use, (4) executing such actions, and (5) maintaining this lower level of use over some period of time. This multi-step process requires mental, physical, and additional resources, and must be repeated for each DR event. There may be benefits to this repetition: previous work has found that people look to their own past pro-environmental actions as a signal of their own environmental identities, potentially resulting in positive spill-over to other behaviors (Van der Werff et al., 2014). Because voluntary curtailment provides people with many opportunities to engage in energy conservation efforts, it may foster environmental identity and lead to performance of other environmentally beneficial behaviors.

To maximize impact, however, it is also important to consider longevity of savings and accuracy in curtailment forecasting (i.e., efforts to predict the magnitude and temporal and geographic distribution of load reduction for upcoming DR events, which are critical for maintaining power reliability). Because people often have inaccurate perceptions about the impacts they can make with various energy conservation behaviors (Attari et al., 2010), leaving curtailment choices to consumers may result in smaller or less reliable reductions than a direct control approach, even among motivated consumers. On the other hand, while direct control systems permit less consumer choice, they may be associated with lower variability in curtailment levels, thereby improving curtailment forecasts. Under many direct control programs, participating in DR events is the default choice, eliminating the need for consumers to repeatedly go through the previously described process, and often requiring no action at all on the part of end-users. Research from the field of Behavioral Economics has demonstrated that people are significantly more likely to select default options (Johnson and Goldstein, 2003), including those related to electric power (Pichert and Katsikopoulos, 2008), and direct control DR systems leverage this principle. Additionally, in simplifying the load curtailment process via automation, direct control programs also require less ongoing effort of end-users, which could potentially support savings over longer periods of time.

Despite the strength of direct control programs in achieving reliable reductions, their appeal can be marred by privacy and autonomy concerns. Among the most notable concerns are perceptions that utilities can use smart grid technologies to (1) directly control a variety of home equipment without consumer permissions or opt-out options; and (2) infer specific behaviors in which occupants are engaging, such as cooking or eating (Krishnamurti et al., 2012; Hess, 2014). In a similar vein, a recent study found that consumers preferred the option of choosing how to curtail consumption to direct control technologies (Leijten et al., 2014). These finding are in alignment with the TPB, which states that perceived control is an important predecessor of behavior. Accordingly, direct control programs that do not foster a sense of control will likely have lower program enrollment compared to DR programs that do so.

To gain greater acceptance, direct control systems should cultivate a sense of consumer control. This could potentially be accomplished by providing some level of consumer choice. What may be indicated is a flexible control strategy, allowing consumers to retain control of home equipment while also maintaining the accuracy of load predictions via default settings that maximize curtailment. This can be achieved by developing systems that allow for consumer override, flexibility in curtailment levels, and other consumer adjustments; these parameters should also be accounted for in curtailment forecast models. It is equally important that consumers recognize that they can adjust such systems—and that participation benefits the environment. For some consumers, however, voluntary curtailment may remain a more attractive option. Identifying moderating variables that differentiate the impacts of types of consumers, DR strategies, and contextual influences on technology adoption is a growing area to which behavioral science can contribute.

Flexible control DR strategies may offer one path forward, but program enrollment represents a significant barrier to participation, and overcoming this barrier is not trivial. Current DR program participation rates are estimated at less than 10%, and actual compliance rates are likely lower (United States Federal Energy Regulatory Commission, 2009). Achieving the load reduction objectives of the coming decades will require increasing levels of consumer engagement. Toward this end, utility-consumer connectivity must be enhanced. For instance, it has been recommended that programs shift from a one-way, utility-to-consumer approach to a more interactive relationship (Vine et al., 2013). Using CBSM to identify motivators for program participation, and building these into recruitment strategies, could boost enrollment rates.

### Time-of-use Pricing

Another smart grid tool that can reduce peak load is variable pricing plans. For instance, time-of-use (TOU) pricing plans aim to discourage energy use during peak times of the day by charging more during high-use periods (typically mid-afternoon hours) and less during off-peak hours. Under TOU programs, usage tends to shift to off-peak times, but the total amount consumed generally remains consistent (Lutzenhiser et al., 2007). By applying financial incentives, these programs invoke operant stimulus control to reduce consumers’ peak energy use, specifically by punishing (with higher prices) on-peak use and reinforcing (with lower prices) off-peak use (Skinner, 1953). A large body of research has shown that reward can be effective in promoting behavior change, especially while incentives are in place, and reward have been effective in reducing home energy consumption below baseline use levels (Hayes and Cone, 1977; Walker, 1979; Winett et al., 1979; McClelland and Cook, 1980) as well as below levels of information-only and control groups (Winett et al., 1979; Midden et al., 1983).

Despite the potential for reward to reduce demand, energy savings associated with reward have been shown to wear off (McClelland and Cook, 1980) and even to rebound after reward are withdrawn (Walker, 1979). For TOU pricing, if off-peak price breaks cannot be sustained long-term, energy loads typically return to pre-TOU pattern. This effect has been observed across a variety of behaviors, including recycling (Wang and Katzev, 1990), hand washing among healthcare workers (Pareira das Neves et al., 2004), and smoking cessation (Donatelle et al., 2004), and may suggest behavioral habituation. One promising alternative can be found in Self-Determination Theory, which suggests that providing reward for behavior that might otherwise occur through intrinsic motivation can weaken intrinsic motives, and may ultimately reduce the performance of the target behavior (Deci and Ryan, 1985). In other words, reward can be counterproductive over the long-term if they undermine intrinsic motivation to act.

Combining reward with other behavior modification strategies in a way that facilitates transition of the contingency from external reward to internal factors may be a more effective long-term strategy. For example, one approach is to identify underlying values as indicated by the Value-Belief-Norm Theory. CBSM offers a vehicle for identifying these values and developing an intervention with which they resonate. Such interventions have been found to be more effective in promoting pro-environmental intentions than simple information alone (Bolderdijk et al., 2013). Historically, utilities have relied heavily on financial incentives to drive consumer behavior, but this is slowly changing with availability of newer technologies that leverage other principles of behavior change. Given the cost of incentives and their potential to undermine long-term goals, we recommend that reward be applied to one-time actions or to behaviors that are performed infrequently, rather than recurring actions.

### Energy Feedback

The proliferation of smart electric meters, most of which record energy data in intervals of one hour or less, has greatly expanded the possibilities for partnering with consumers. First, providing immediate feedback mitigates the issue that people are generally more responsive to immediate rather than future consequences, which arises from the fact that most consumers pay for energy long after using it (Frederick et al., 2002). Smart meter data can be made available in near real-time to consumers through a variety of platforms, including websites, mobile phones, and in-home displays, enabling consumers to connect their behavior with its consequences. The more granular energy data has enabled utilities to advance from providing energy feedback as part of monthly (or even annual) billing to providing near real-time data that can enhance usability and relatability.

Utilities generally view this feedback as a form of education, but evidence from behavioral science shows that feedback can be a very powerful tool for changing behavior. Studies suggest that personalized feedback can produce significantly more energy savings than merely providing educational materials about household energy use (Seligman and Darley, 1977; Midden et al., 1983; Hutton et al., 1986). In addition, smart meters offer higher resolution feedback, which has been found to produce greater levels of energy conservation (Ehrhardt-Martinez et al., 2010), highlighting this potential of the smart grid to support energy efficiency.

Energy feedback represents one type of feedback, but with energy data of entire consumer bases, utilities can also provide feedback about the performance of others, thereby conveying normative information. A growing body of research has shown that descriptive normative feedback—information about what others are doing—can be associated with behavior change (Cialdini et al., 1990; Schultz et al., 2007; Goldstein et al., 2008; Nolan et al., 2008; Abrahamse and Steg, 2013). Under the Theory of Planned Behavior, Norm Activation Model, Value-Belief-Norm Theory, and Focus Theory of Normative Conduct, this may occur through enhancing normative beliefs in support of conservation. In addition, as per the Focus Theory of Normative Conduct, combining descriptive normative feedback with an injunctive message—feedback that conveys social approval—can mitigate the undesirable “boomerang” effect that arises when an individual increases use after receiving feedback that others are consuming more. For instance, Schultz et al. (2007) found that among households using less energy than average at baseline, those who received descriptive normative feedback only increased their use, but this effect was attenuated among those who also received injunctive feedback (in this case, a smiley face affirming lower use than average). This is a relatively new area of research, but findings suggest that building social tools into the delivery of energy data offers considerable promise in efforts to improve energy efficiency. Refinement of social feedback tools requires a better understanding of several potential moderators: type of social feedback, household characteristics (e.g., household size), sociodemographic considerations such as income, and psychosocial factors such as group cohesion (Abrahamse and Steg, 2011, 2013).

### Disaggregation Technologies

Moving beyond household-level feedback, technologies that provide energy feedback at the appliance level are coming to market. One option is through smart appliances, which monitor and report their level of consumption, but which are often cost-prohibitive. Another option is non-intrusive load monitoring, which disaggregates the household energy signal into individual appliance loads. Non-intrusive load monitoring is only possible with high-resolution consumption data such as that provided by smart grid technologies.

The level of specificity offered by appliance feedback marks a significant innovation from whole-house feedback, which, while useful when compared to on-bill feedback, falls short of providing information on specific behaviors consumers can undertake to conserve. Household-level feedback still requires consumers to generate a mental list of what is using energy in their home, which can be overwhelming and ultimately inhibit action. Eliminating the need for this process, appliance-level feedback instead informs consumers of exactly which appliances are consuming energy, enabling them to associate discrete behaviors with energy (and sometimes cost) impacts. Disaggregation can also offer a straightforward action step, which may lead to an enhanced sense of competence or perceived control, as suggested by Self-Determination Theory and Theory of Planned Behavior, respectively.

Combined with specific recommendations for improved efficiency and conservation, disaggregated feedback is a promising strategy. However, to date, few studies have evaluated the effectiveness of such technologies on load-shifting and conservation, in part because such systems are so new. Future research in this area is needed.

### Smart Automation

Some smart appliances such as thermostats and dishwashers offer more than just appliance-level feedback; they also offer scheduling capabilities and DR signal automation (the ability to be directly controlled by utilities). Technologies such as Internet-enabled programmable thermostats are outfitted to dovetail with direct control DR strategies to curtail peak loads, in addition to offering conservation potential. These technologies can function as “set and forget,” requiring minimal ongoing effort on the part of the end-user after initial device purchase, installation, and set-up. As mentioned, research suggests that the conservation potential of efficiency technologies is greater than that of curtailment approaches (Gardner and Stern, 2008). Automation removes the need to sustain behavior change over time, reducing end-user burden and increasing predictability of curtailment outcomes, which is important for improving the accuracy of demand forecasting and supporting power reliability.

However, it is also important to point out that effort is only one of several important factors predicting adoption and optimal use of smart automation and other efficiency technologies. Behavioral science can help address additional challenges in technology design and adoption. For instance, in line with the Theory of Planned Behavior and Self-Determination Theory, devices should foster a sense of control and autonomy, for instance, with user-friendly designs and ease of operation. Similarly, for products that permit utility control, flexible default and remote control settings that allow for consumer modifications should be developed (see Demand Response section above).

### Electric Vehicles

Electric vehicles (EVs) offer potential for supporting grid reliability. Specifically, vehicle battery technologies that discharge energy back into the grid during high usage periods offer potential for distributed storage networks and a fundamentally new strategy for managing peak demand. In such a system, AMI technology collects data on vehicle charging schedules, which can be used to generate intelligent, automated charging and discharging schedules that dynamically accommodate grid-wide demand fluctuations. Because each vehicle battery has relatively low storage capacity, widespread consumer adoption is a necessity for making this possible. Globally, less than 1% of light-duty passenger vehicles are EVs (Trigg and Telleen, 2013). For EVs to plug into the smart grid as a viable distributed storage technology, using behavioral science to increase consumer adoption of EVs, as well as enrollment and optimal participation in charge–discharge programs, are critical.

As an emerging technology, EVs face financial, technical, and social barriers to broader consumer acceptance. Perceived costs, including financial and convenience, are among the strongest barriers to adoption (Bockarjova and Steg, 2014). Because purchasing a car tends to be a relatively infrequent behavior, the use of financial incentives, such as government subsidies and tax breaks, is likely to be helpful for increasing EV adoption. The availability of financial incentives is positively correlated with EV adoption rates, but price signals represent only one predictor of EV adoption (Bockarjova and Steg, 2014; Sierzchula et al., 2014). Even among consumers with favorable attitudes toward EVs, reduced range and long charging times are among the top concerns, and many consumers report unwillingness to compromise on these features (Ewing and Sarigöllü, 2000; Hidrue et al., 2011). However, the same consumers are willing to pay high, up front premiums for EVs with longer ranges and faster charging capabilities (Hidrue et al., 2011), highlighting that price breaks alone are not sufficient to increase adoption rates. Symbolic attributes, which signal the impact of a belonging on one’s identity and social status, have also been identified as a key factor underlying EV purchase, over and above practical considerations such as cost and range (Heffner et al., 2007; Noppers et al., 2014). Campaigns that tap into these identity concerns may contribute to higher adoption rates.

Diffusion of Innovations theory suggests that social influence also plays an important role in the adoption of new technologies (Rogers, 2003). Under this model, the first 2.5% of individuals to adopt a technology (Innovators) tend to rely on technical information, followed by the Early Majority, who incorporate the opinions of others in their decision-making about new technologies. More Early Majority individuals will be acquiring EVs as the EV market share expands, and therefore harnessing the power of social influence is indicated. Recent research has demonstrated the success of social influence in promoting engagement in a variety of sustainable behaviors, but most of these studies have focused on changing low-cost, habitual behaviors (Schultz et al., 2007; Goldstein et al., 2008; Nolan et al., 2008). Energy efficiency technologies such as EVs involve one-time or infrequent behaviors and high up-front costs, yet offer long-term energy conservation potential and require minimal ongoing effort from consumers. Research is needed to investigate whether social influence can effectively be used as a tool of persuasion in such contexts.

Another consideration is that drivers may object to having their batteries drained during high-use periods, a barrier to charge–discharge programs. As with direct control DR and smart automation, it is essential that flexible rules be developed to permit some consumer control in charge–discharge programs, and that consumers retain a sense of control. Using approaches such as CBSM to uncover additional barriers and motivators to participation in such programs will be critical in crafting strategies to increase enrollment.

### Solar Panels

By offering on-site, distributed generation, the excess of which can be routed to overstressed portions of the grid, residential solar panels fit into the smart grid by offering another strategy to boost grid reliability. Currently, however, solar accounts for less than 5% of energy generated in the United States (United States Energy Information Administration, 2015b). As with EVs, for solar to be a viable distributed generation option, consumers must adopt the technology on a considerably wide scale; because solar panels offer long-term savings without ongoing consumer efforts, increasing installations is currently a key issue. High up-front costs and technical considerations represent barriers to this being a reality. Financial incentives may be well-suited to increasing residential solar installations, but alone will not address all barriers to adoption. For instance, recent findings suggest that social influence plays an important role in the installation of rooftop solar systems. Described as the “solar contagion” effect, studies have found that adding a solar system, which is usually visible to passersby, to a single home in a neighborhood significantly increases the average number of installations within a half-mile radius (Bollinger and Gillingham, 2012; Graziano and Gillingham, 2014). As per the Theory of Planned Behavior, Norm Activation Model, Value-Belief-Norm Theory, and Focus Theory of Normative Conduct, a social influence approach like this can strengthen normative beliefs in support of solar panel installation, and contribute to elevated adoption rates. In addition, solar panels are often very visible features of a home, perhaps conveying to others something about the occupants’ identities and/or social status. Behavioral science should identify potential symbolic attributes of solar systems, as tapping into these may also support higher adoption rates.

## Conclusions and Future Directions

In summary, behavioral science can play an important role in unlocking the potentials of smart grid technologies to reduce overall energy consumption, curtail peak demand, and expand distributed storage and generation options. There is a growing body of research focused on the behavioral aspects of energy consumption, and findings from this research can be overlaid on programs that leverage the emerging smart grid infrastructure. As reviewed in this paper, behavioral science is already being used and can be further leveraged to improve DR programs, time-of-use pricing, energy use and disaggregated feedback, smart automation, and distributed storage and generation options through EVs and solar panels.

In this review, we described how different theories can be used to explain the adoption and use of different smart grid technologies. As has been pointed out previously in relation to other environmentally-relevant behaviors (Huijts et al., 2012; Perlaviciute and Steg, 2014), we believe there is value in developing a more integrated approach to explain the acceptance, adoption, and use of smart grid technologies. Such a framework can guide researchers and practitioners in the application of relevant theories to varying contexts, technologies, consumer characteristics, and behaviors. For instance, such a framework could aid in understanding, and potentially facilitating, spill-over effects: how does adopting and/or using one smart grid technology translate to the adoption and/or use of others? A recent study based on the Norm Activation Model suggests that general awareness of the impact of energy use on the environment, belief that one can mitigate these impacts, and a sense of moral obligation to do so can motivate a variety of energy reduction behaviors (Van der and Steg, 2015). In addition, because different factors appear to foster adoption and use of different smart grid technologies, it is also important to identify the role of moderators on several levels: household characteristics, sociodemographic variables, and psychosocial variables (Abrahamse and Steg, 2011, 2013). Segmenting consumers to identify what technologies resonate best with whom, in what situations, can maximize savings.

A central consideration in partnering with consumers in the smart grid relates to persistence of behavior, which influences energy savings and power reliability. Available data show that effects of behavioral curtailment strategies tend to taper off over time, leading to questions about the long-term value of these strategies. On the other hand, efficiency strategies such as direct control DR, smart automation, EV adoption, and solar panel installation are not subject to the same limitations. How to move consumers past the higher up-front costs, privacy/autonomy concerns, and technical barriers commonly associated with efficiency technologies is a key question for behavioral scientists. We advocate for flexible control strategies that involve utility-set defaults and remote control options (e.g., smart appliances in DR direct control and EVs in charge–discharge programs), while also allowing consumers the freedom to modify these settings.

In addition, there is a growing need for rigorous program evaluations, publicized results, and expanded opportunities for utilities and behavioral scientists to connect. Future work should investigate outcomes beyond kWh savings to explore underlying processes of behavior change. Findings from this work will offer further insights for optimizing the potentials of the smart grid.

### Conflict of Interest Statement

The authors declare that the research was conducted in the absence of any commercial or financial relationships that could be construed as a potential conflict of interest.
